# Comparison of Intra-Arterial Chemotherapy Efficacy Delivered Through the Ophthalmic Artery or External Carotid Artery in a Cohort of Retinoblastoma Patients

**DOI:** 10.3389/fmed.2021.658305

**Published:** 2021-06-11

**Authors:** Shichong Jia, Xuyang Wen, Min Zhou, Xiaoyu He, Minglei Han, Jiayan Fan, Renbing Jia, Xianqun Fan

**Affiliations:** Department of Ophthalmology, Shanghai Key Laboratory of Orbital Diseases and Ocular Oncology, Shanghai Ninth People's Hospital, Shanghai JiaoTong University School of Medicine, Shanghai, China

**Keywords:** retinoblastoma, intra-arterial chemotherapy, external carotid artery, ophthalmic artery, middle meningeal artery

## Abstract

**Purpose:** To evaluate the efficacy of an external carotid artery (ECA) alternative route in intra-arterial chemotherapy (IAC) for treatment of retinoblastoma.

**Methods:** In this retrospective, single-centre, case-control study, 98 retinoblastoma patients who received successful IAC were included. The drug delivery routes were the primary ophthalmic artery (OA) route and the ECA route when OA catheterization was not feasible.

**Results:** A total of 337 successful IAC procedures were performed in our study, of which 32 (9.5%) procedures were performed through the ECA route. Eighteen eyes (18.4%) accepted at least one IAC through branches of the ECA. Statistical analysis showed that there was no significant difference in ocular clinical results (enucleation, death, recurrence and event-free) between the ECA and OA routes. No significant association was found between the route of drug delivery and the ocular survival time (*p* = 0.69). The use of ECA catheterization in at least one IAC cycle was not a predictor of enucleation (HR: 1.58; 95% CI: 0.56–4.46, *p* = 0.39). The increasing number of procedures through the ECA route did not increase the risk of enucleation (HR: 1.64; 95% CI: 0.42–6.39, *p* = 0.48).

**Conclusion:** The ECA alternative route did not affect the efficacy of IAC in retinoblastoma. When the standard OA approach is not feasible, ECA system catheterization should be considered.

## Introduction

Retinoblastoma is the most common primary intraocular cancer in children, and it has been estimated that the annual incidence of retinoblastoma is 264 cases in North America, 464 cases in Europe and 4,258 cases in Asia (1, 2). The number of cases in China ranks first in the world, and more than 70% of the diagnosed eyes present at advanced stages (3). In the past three decades, treatment for retinoblastoma has undergone a shift from radiotherapy to systemic chemotherapy, and the development of *in situ* chemotherapy has greatly improved global salvage (1, 4). Intra-arterial chemotherapy (IAC) is one of the best advances in *in situ* chemotherapy, which efficiently increases the local intraocular drug concentration and reduces the systemic side effects of intravenous chemotherapy (IVC). The conventional mode of anticancer drug delivery in IAC is through infusion into the ophthalmic artery (OA) (5). However, in some cases, IAC may fail because of difficulties with angiographic visualization or catheterizing on OA (6). Hence, it is necessary to find an alternative route for drug delivery when OA catheterization is difficult. The middle meningeal artery (MMA), originating from the maxillary artery (MA) of the external carotid artery (ECA) system, has been reported as one of the most commonly used alternative approaches (7). In this article, we reviewed the IAC treatment experiences of 98 retinoblastoma patients at our center and compared the clinical outcomes of the standard OA route and alternative ECA route to verify whether there is a difference in therapeutic effect between these two routes.

## Methods

### Patients

The subjects of this retrospective study included patients diagnosed with unilateral or bilateral intraocular retinoblastoma from January 2016 to December 2019 in whom IAC was successfully performed. The exclusion criteria were as follows: (1) anterior chamber invasion, uvea, sclera or optic nerve infiltration, intracranial metastatic disease and secondary glaucoma at diagnosis; (2) a follow-up period of <6 months; and (3) incomplete data collection. This research was approved by the Ethics Committee of Shanghai Ninth People's Hospital affiliated to Shanghai JiaoTong University School of Medicine. The patient's medical records and fundus photography at the time of diagnosis were retrospectively analyzed to obtain demographic data.

### Protocol

With the consent of parents or legal guardians, IAC was performed in the operating room under endotracheal intubation and general anaesthesia. A 4F pediatric guide microcatheter (diameter 0.97 mm) was used to puncture the femoral artery. The sheath was subsequently positioned and the internal carotid artery (ICA) supplying the affected eye was inserted. Serial angiography runs were performed to observe the anatomy of the OA. Then, super-selective Prowler 10 microcatheter (diameter 0.57 mm) was introduced to an ostial position, and chemotherapeutic drugs were injected into the OA. Drug regimens are shown in Supplementary Table 1. The time for drug infusion was 20 to 25 min. Final lateral arteriography of the ICA or the common carotid artery (depending on the route of administration) was conducted immediately after surgery to exclude complications such as vasospasm, embolism or dissection. After treatment, the children who recovered from anaesthesia were observed for 4–6 h and discharged on the next day.

OA was always the first choice. If OA could not be catheterized directly or an adequate choroidal blush was unattainable, we adopted the alternative ECA branch approach according to the anatomical condition. During the treatment, intravitreal chemotherapy was applied for the control of vitreous seeds. Commonly used drug for intravitreal injections was melphalan (20–50 μg per injection) or topotecan (20–50 μg per injection), and the dose was dependent on tumor volume. Local treatments, like photocoagulation and cryotherapy, were also applied for consolidation after IAC cycles.

### Groups and Outcome Variables

The treated eyes were retrospectively divided into two groups. The first group consisted of eyes that received IAC merely via the selective OA route, whereas the other group included eyes that received treatment using at least one cycle of alternative ECA approach. Primary outcome was enucleation. Death, recurrence and event-free survival were also analyzed. Recurrence was defined as new tumor activity, including regression of tumors or new subretinal seeds, requiring retreatment more than 3 months after completing IAC treatment. Event-free status was defined as eye-preservation status without uncontrollable tumors, recurrence, metastases or deaths, by the end of follow-up.

### Statistical Analysis

Categorical variables were reported as frequencies (percentages), whereas normally distributed continuous variables were presented as the mean (standard deviation), and variables without a normal distribution were reported as the median (quartile). We analyzed categorical variables by λ^2^ tests. For normally distributed continuous variables, Student's *t*-tests were applied, while Mann-Whitney tests were used for highly skewed variables. The Kaplan-Meier method was used to evaluate ocular survival time, in which events were defined as enucleation. The log-rank test was applied to determine whether there was statistical significance. The ocular survival probability was evaluated by Cox proportional hazard model and shown by HR (95% CI). The covariates used to adjust HR were those with *p* < 0.1 in the univariate model and those that may influence the outcome. For all tests, a value of *p* < 0.05 was considered statistically significant. All analyses were performed with SPSS (version 22.0, IBM, Armonk, NY) and R version 3.6.1 (The R Foundation).

## Results

### Patients

In our study, 98 eyes in 96 retinoblastoma patients received 337 successful IAC sessions overall. The median age at diagnosis was 21.62 months, and 46.9% of the retinoblastomas occurred in male patients; 75.5% of the study cases were unilateral disease. Each eye received a median of 4 IAC cycles, ranging from 1 to 9. Among all 98 eyes, 7 eyes (7.1%) were in group C, 27 eyes (27.6%) were in group D, and 64 eyes (65.3%) were in group E. Fifty-one (52.0%) eyes which previously accepted IVC changed their treatment to IAC because of poor tumor control under IVC. Forty-three eyes (43.9%) received intravitreal chemotherapy (median: 2; range: 1–5), among which 1 eye was in group C, 17 eyes were in group D, and 25 eyes were in group E. Thirty (30.6%) eyes received local treatment for consolidation after IAC cycles: 22 (20.6%) eyes received photocoagulation (median: 1; range: 1–6) while 20 eyes (20.4%) received cryotherapy (median: 2; range: 1–5).

**Figure 1:**
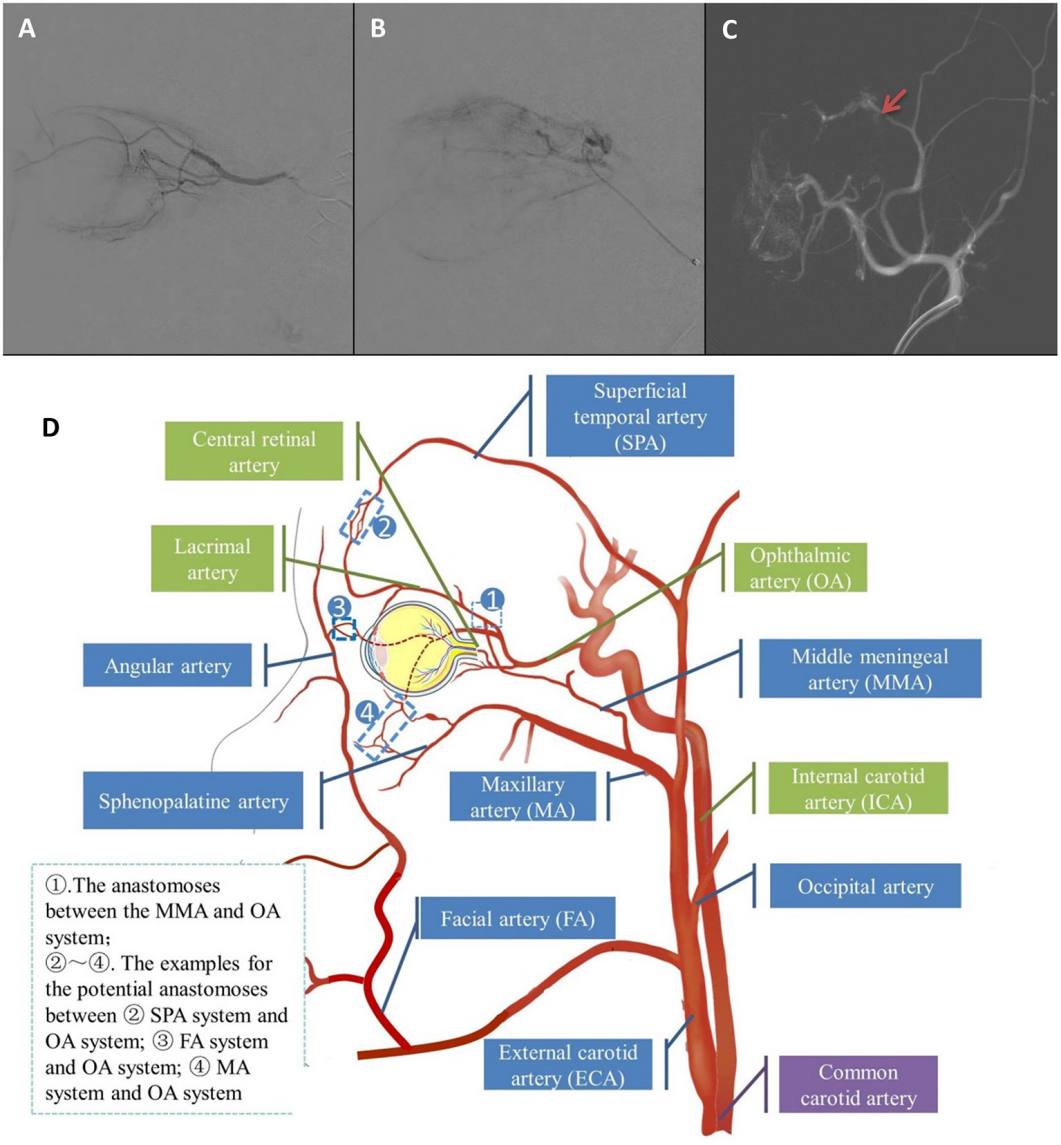
**(A)** Representative angiography of direct OA catheterization. **(B,C)** Representative angiography of alternative MMA approach catheterization (Red arrow showed where the catheter was advanced). **(D)** Schematic illustration of the branches of OA and ECA and their several potential anastomoses.

ECA group consisted of 18 eyes (18.4%) that received at least one ECA catheterization (Figures 1B,C), while OA group consisted of the remaining 80 eyes (81.6%) which were treated with OA catheterization (Figure 1A). A total of 32 procedures (9.5%) in 337 IAC sessions were performed via the ECA route. The causes for the adoption of ECA routes were temporary vasospasm of OA in seven eyes, OA occlusion in eight eyes and OA variation in three eyes. In the ECA group, the median ECA catheterization was 1.5 cycles, ranging from 1 to 5 cycles. One cycle of ECA catheterization was performed in half of eyes in ECA group, while 2 cycles in 6 eyes, 3 cycles in 2 eyes and 5 cycles in 1 eye. No MMA thrombosis happened in the IAC cycles through ECA catheterization. The demographic and clinical features of the two groups showed no significant difference (Table 1).

**Table 1:** The demographic and clinical features of eyes receiving IAC.

	**Total**	**OA group**	**ECA group**	***P*-value**
	**(*N* = 98, 100%)**	**(*N* = 80, 81.6%)**	**(*N* = 18, 18.4%)**	
Age at diagnosis (m)	21.62	21.07	25.60	0.59
	(10.08, 32.46)	(9.36, 32.63)	(11.64, 29.59)	
Gender				0.20
Male	46(46.9%)	40(50.0%)	6(33.3%)	
Female	52(53.1%)	40(50.0%)	12(66.7%)	
Laterality				0.23
Unilateral	74(75.5%)	58(72.5%)	16(88.9%)	
Bilateral	24(24.5%)	22(27.5%)	2(11.1%)	
Side				0.77
Left	52(53.1%)	43(53.8%)	9(50.0%)	
Right	46(46.9%)	37(46.3%)	9(50.0%)	
IIRC Group				0.19
C	7(7.1%)	6(7.5%)	1(5.6%)	
D	27(27.6%)	25(31.3%)	2(11.1%)	
E	64(65.3%)	49(61.3%)	15(83.3%)	
Previous IVC				0.48
Yes	51(52.0%)	43(53.7%)	8(44.4%)	
No	47(48.0%)	37(46.3%)	10(55.6%)	
Intravitreal chemotherapy				
Yes	43(43.9%)	34(42.5%)	9(50.0%)	
No	55(56.1%)	46(57.5%)	9(50.0%)	0.56
Photocoagulation				0.23
Yes	22(22.4%)	16(20.0%)	6(33.3%)	
No	76(77.6%)	64(80.0%)	12(66.7%)	
Cryotherapy				0.11
Yes	20(20.4%)	19(23.8%)	1(5.6%)	
No	78(79.6%)	61(76.3%)	17(94.4%)	
No. of IAC cycles	4(2.75,4)	4(2,4)	4(3,4)	0.16

*OA, ophthalmic artery; ECA, external carotid artery; IAC, intra-arterial chemotherapy; IIRC, intraocular international retinoblastoma classify; IVC, intravenous chemotherapy*.

### Ocular Survival

Overall, the rates of enucleation in the OA group and ECA group were 33.8 and 27.8%, respectively, showing no significant difference (*p* = 0.63). The following analysis demonstrated no significant difference in the enucleation rates between the two routes in each subgroup (Group C, D or E; unilateral or bilateral; with previous IVC or not) (Table 2). Among the eyes that received the different number of procedures through the ECA route, the enucleation rates showed no significant difference (*p* = 0.16).

**Table 2:** The outcomes of 98 eyes receiving IAC.

		**Enucleation**			**Event free**			**Recurrence**		
		**OA group**	**ECA group**	***P*-value**	**OA group**	**ECA group**	***P*-value**	**OA group**	**ECA group**	***P*-value**
Total		27(33.8%)	5(27.8%)	0.63	45(56.3%)	9(50.0%)	0.63	12(15.0%)	4(22.2%)	0.49
IIRC Stage	C (*n* = 7)	0(0.0%)	0(0.0%)	-	6(100%)	1(100%)	-	0(0.0%)	0(0.0%)	-
	D(*n* = 27)	1(4.0%)	0(0.0%)	1.00	19(76.0%)	2(100%)	1.00	5(20.0%)	0(0.0%)	1.00
	E(*n* = 64)	26(53.1%)	5(33.3%)	0.18	20(40.8%)	6(40.0%)	0.96	7(14.3%)	4(26.7%)	0.27
Laterality	Unilateral(*n* = 74)	25(43.1%)	4(25.0%)	0.19	31(53.4%)	8(50.0%)	0.81	4(25.0%)	7(12.1%)	0.24
	Bilateral(*n* = 24)	2(9.1%)	1(50.0%)	0.24	14(63.6%)	1(50.0%)	1.00	5(22.7%)	0(0.0%)	1.00
Previous IVC	No(*n* = 51)	15(40.5%)	2(20.0%)	0.29	20(54.1%)	5(50.0%)	1.00	7(18.9%)	2(20.0%)	1.00
	Yes(*n* = 47)	12(37.5%)	3(27.9%)	0.68	25(58.1%)	4(50.0%)	0.71	5(11.6%)	2(25.0%)	0.30
No. of ECA	0(*n* = 80)	27(33.8%)		0.16	45(56.3%)		0.40	12(15.0%)		0.63
	1(*n* = 9)	1(11.1%)			4(44.4%)			3(33.3%)		
	2(*n* = 6)	4(66.7%)			2(33.3%)			1(16.7%)		
	3(*n* = 2)	0(0.0%)			2(100.0%)			0(0.0%)		
	5(*n* = 1)	0(0.0%)			1(100.0%)			0(0.0%)		

*OA, ophthalmic artery; ECA, external carotid artery; IAC, intra-arterial chemotherapy; IIRC, intraocular international retinoblastoma classify; IVC, intravenous chemotherapy*.

**Figure 2:**
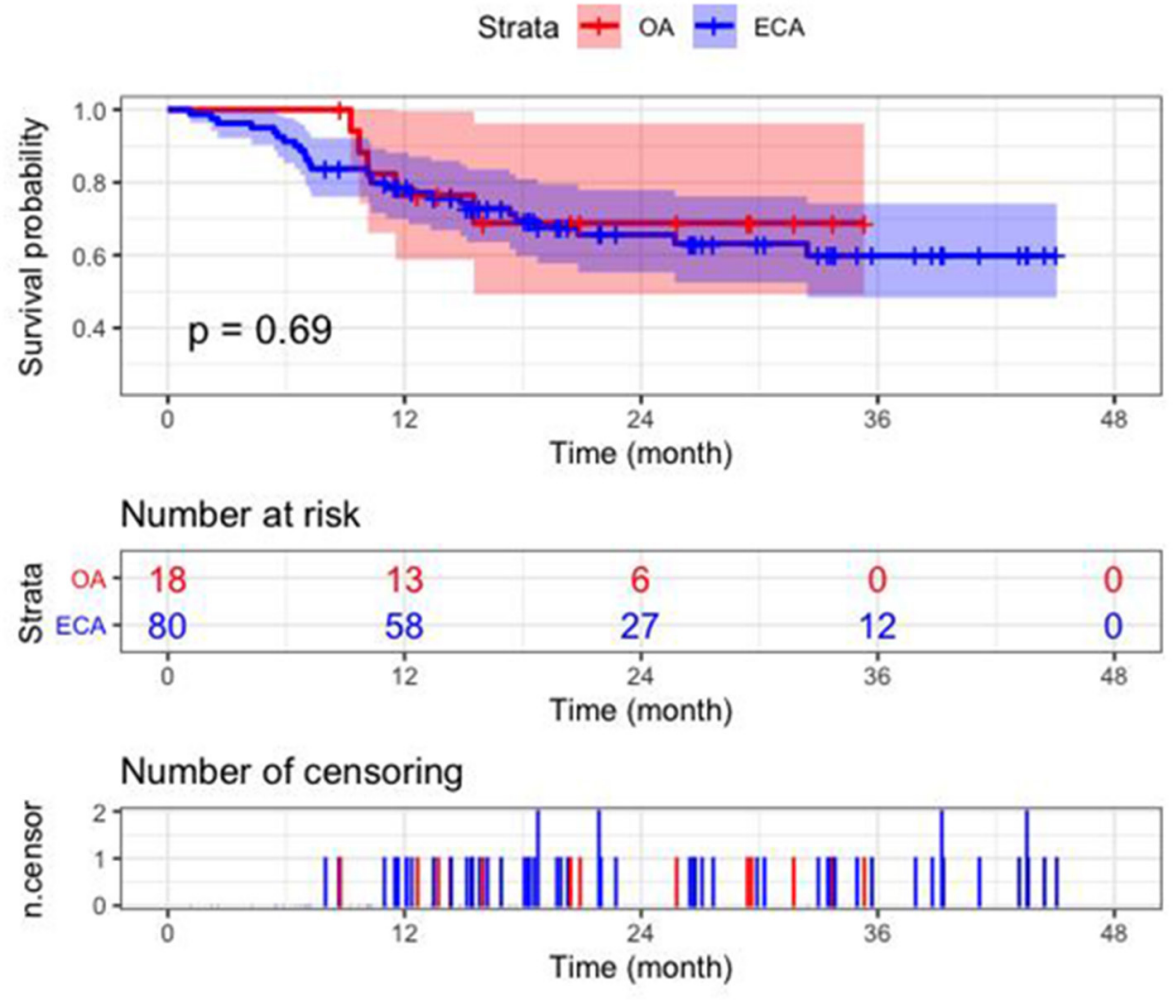
Kaplan–Meier estimates of ocular survival in the OA group and ECA group.

In this study, the globe preservation rates of the OA group were 78.7% (1-year) and 68.8% (2-year), while the 1-year and 2-year eye conservation rates in the ECA group were 77.8 and 77.2%, respectively. The Kaplan-Meier curve showed that no significant association was found between the route of drug delivery and the ocular survival time (*p* = 0.69) (Figure 2). Multivariate Cox proportional hazards model showed that IIRC group E was the only predictor of enucleation (HR: 20.97, 95% CI: 2.77–158.48, *p* = 0.003). The use of ECA catheterization in at least one IAC cycle was not a predictor of enucleation (HR: 1.58; 95% CI: 0.56–4.46, *p* = 0.39). The increase in the number of IAC cycles is significant for globe preservation (HR: 0.70, 95% CI: 0.52–0.93, *p* = 0.015). Other variables did not show any statistically significant results (Table 3). In following analysis of ECA group, the increasing number of procedures through the ECA route was not a predictor of enucleation (HR: 1.64; 95% CI: 0.42–6.39, *p* = 0.48).

**Table 3:** Multivariate Cox proportional hazards model for predicting enucleation.

**Variable**	**Reference**	**HR**	**95% CI**	***P***	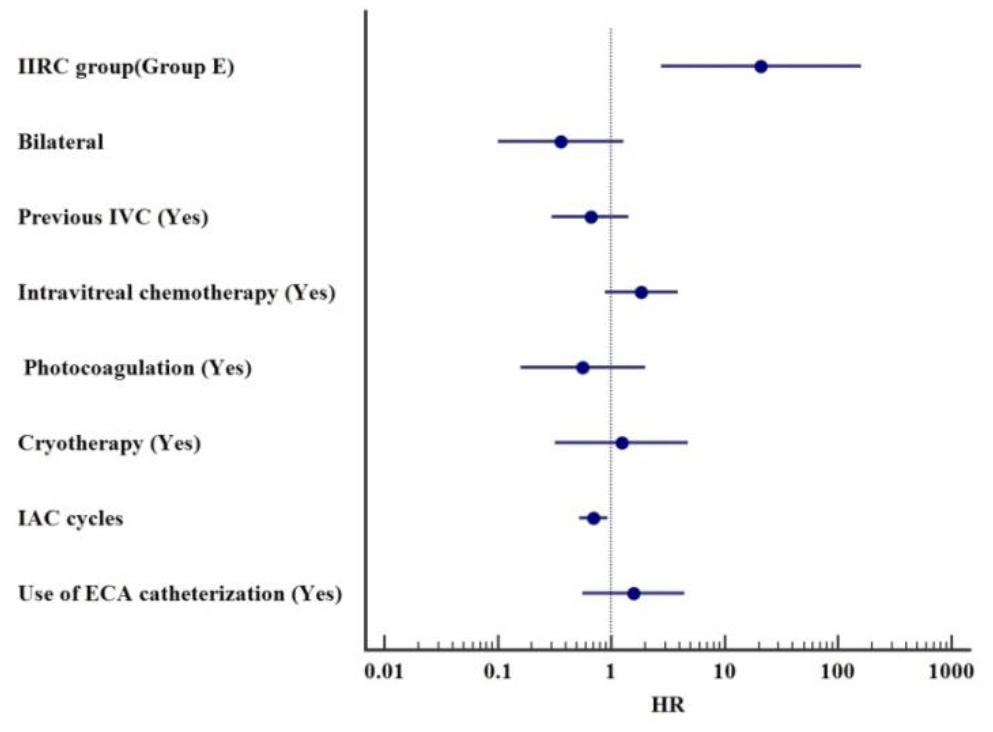
IIRC group (Group E)	Versus Group D	20.97	2.77–158.48	0.003	
Bilateral	Versus Unilateral	0.36	0.10–1.29	0.12	
Previous IVC (Yes)	Versus No	0.66	0.30–1.43	0.29	
Intravitreal chemotherapy (Yes)	Versus No	1.84	0.88–3.86	0.11	
Photocoagulation (Yes)	Versus No	0.56	0.16–1.99	0.37	
Cryotherapy (Yes)	Versus No	1.24	0.32–4.79	0.76	
IAC cycles	One cycle increment	0.70	0.52–0.93	0.015	
Use of ECA catheterization (Yes)	Versus No	1.58	0.56–4.46	0.39	

*ECA, external carotid artery; IAC, intra-arterial chemotherapy; IIRC, intraocular international retinoblastoma classify; IVC, intravenous chemotherapy*.

### Death, Recurrence and Event-Free

In the follow-up, three patients in the OA group developed distant metastases (the metastasis locations were bone, brain and systemic dissemination, respectively) and died of disease, while no distant metastases and deaths occurred in the ECA group. There were 4 cases of recurrence in the ECA group and 12 cases in the OA group, which showed no significant difference (*p* = 0.49). There was no significant difference in the recurrence rate between the two catheterization routes in each subgroup (Table 2). In the ECA group, nine cases exhibited event-free survival, while 45 cases in the OA group were event-free (50.0 vs. 56.3%, *p* = 0.63). The event-free rates in different patients of the two groups were similar (Table 2).

### Complications

The complications reported with IAC include systemic and ocular complications. For myelosuppression in the OA group, there were 23 grade 1, 9 grade 2 and 1 grade 3 myelosuppression cases, while there were 8 grade 1 and 2 grade 2 myelosuppression cases in the ECA group. The occurrence of myelosuppression was not associated with any particular drug delivery route (*p* = 0.59). There was no significant difference in the occurrence of digestive complications such as vomiting and diarrhoea (six in the OA group vs. 1 in the ECA group, *p* = 1.00). Among the ocular complications, the patients in the ECA group had a higher proportion of cataracts (1 in the OA group vs. 3 in the ECA group, *p* = 0.02). Other ocular complications included 3 erythema, 5 ptosis and 17 retinal bleeding events in the OA group and 2 erythema and 7 retinal bleeding events in the ECA group, showing no statistically significant differences.

### Radiation

The radiation parameters (dose–area product (DAP) and fluoroscopic time) were significantly lower when taking the OA approach (571.3 ± 413.6 cGy cm^2^; 6.3 ± 4.6 min) than MMA (1,633.0 ± 828.7 cGy cm^2^; 14.2 ±7.9 min) (*p* < 0.001).

## Discussion

This report constitutes the largest retrospective series that compares the efficacy of two catheterization routes for IAC in Chinese patients with retinoblastoma. All IAC procedures are performed by a well-trained operator, preventing confounding factors that may be introduced by technical differences between operators. Thirty-two (9.5%) procedures in 18 eyes (18.4%) were performed through the ECA route. Our analysis shows that the ocular survival time in the OA group and ECA group did not show any significant difference, and the increasing number of ECA catheterization was not a predictor of enucleation, proving that the alternative ECA route is as efficient as the classic OA route.

In 1954, Reese et al. first tried IAC for retinoblastoma, and the drug infusion route they chose was ICA on the side of the affected eye (8). In the 1990s, the IAC technique was improved for a better selectivity: distal flow was blocked with balloon inflation after catheterization of the ICA, and the drug infusion was performed at the branch point of OA (9). In 2008, Abramson et al. first reported a technique of truly selective OA catheterization for melphalan infusion, which effectively salvages the eyes that are to be enucleated (5). After 10 years of development, IAC has become a widely accepted therapeutic strategy, and several recent studies (10–12) showed that globe salvage rate has achieved 100% for group C, 79–96% for group D and 33–62% for group E, which is consistent with our study (100, 96.3, and 61.6%). The technological change of advancing the catheter to the ostium of the OA has also greatly improved the safety of IAC (13). However, OA catheterization is not always successful, and several reasons have been reported (14–16).

First, the OA structure may make catheterization impossible or unsteady, such as a diameter that is too small in younger patients or an acute origin of OA from the ICA. Second, in more than 90% of cases, OA originates from the ICA, however in some eyes, there was an anatomic variant, and several possible anastomoses of the ECA may provide alternative pathways for the orbital blood supply; sometimes, the MMA may even replace the OA as the main blood supply artery for the eye (17). Third, previous successful OA catheterization may in turn lead to stenosis or complete occlusion of the OA. Sweid et al. (18) had reported that the number of IAC sessions was an independent predictor of OA occlusion, and the risk would increase more than 3 times with each IAC session. Moreover, temporary vasospasm of OA may also lead to catheterization failure. The introduction of the catheter itself into the origin of the artery may be responsible for this hemodynamic disorder (17). In our cases, over 80% of the eyes in the ECA group experienced previous successful OA catheterization. Among them, some transient vasospasm of OA did not appear at the next cycle, but some eyes suffered from irreversible OA stenosis.

The anatomical basis of the alternative route for IAC lies in the anastomosis between OA and the branches of the ECA. Several potential anastomoses between the OA and the ECA system are illustrated in Figure 1D, and the most common anastomoses among them are connections between the lacrimal artery (LA) and MMA, also known as the orbital branch of the MMA, often located at the orbital apex (19, 20). In our studies, all alternative route catheterizations were performed through MMA. Moreover, the main branches of the ECA, the facial artery, the MA and the superficial temporal artery (SPA), may also have anastomoses with the OA system, distributed in the orbital apex, anterior orbit, and suborbital regions (16). The study of Bertelli et al. (21) also reflects the complexity of the anastomosis between the OA and ECA systems. In their IAC sessions performed through the ECA systems, anastomosis between the angular artery and dorsal nasal artery, SPA and supratrochlear artery, and anterior deep temporal artery and LA was also used.

Some might worry that the indirect drug delivery, rather than direct OA route infusion, may affect the therapeutic effect. However, in our study, there was no statistical difference in the treatment outcome of the two routes. In the previous study, Gobin et al. firstly demonstrated the feasibility of MMA catheterization as an alternative for IAC, but therapeutic effect was not described (22). Klufas et al. reported their results of IAC which performed through alternative routes. Among the 18 eyes that had received at least one MMA catheterization (10 eyes) or balloon occlusion (11 eyes), 94.4% were stable or had varying degrees of tumor control (14). Sweid et al. performed ECA catheterization in 22 cases (3.3%) and ICA balloon in 20 (3%) cases, and they found that the alternative approaches did not increase the probability of eye enucleation [OR = 0.72 (0.17–3.04)]. The main reasons for the failure of OA catheterization were OA stenosis and OA occlusion, accounted for more than 80% of the cases (23). Bertelli et al. (21) have proved that using ECA branches (not only MMA but also several periorbital vessels) for drug delivery is as effective as classical OA route. To sum up, the ECA catheterization provided IAC opportunities for patients who were unable to receive OA catheterization and can also achieve tumor control.

Systemic complications of IAC include myelosuppression and digestive symptoms, and myelosuppression is the most common, but we did not find a relationship between systemic complications and a particular route of drug delivery, as reported in previous literature (21). Most ocular local complications were mild in our study. In the previous study, Guasti et al. (24) found that, compared with ECA branches, the OA approach was associated with significantly lower radiation parameters [fluoroscopy times: ECA (997 ± 732 s) vs. OA (504 ± 354 s); DAP: ECA (1,257 ± 934 cGy cm^2^) vs. OA (526 ± 328 cGy cm^2^)]. The parameters and trends as we reported were in line with this study. Bertelli et al. (21) and Boddu et al. (25) also reported a similar trend, which confirmed the higher radiation exposure in IAC cycles performed through ECA branches. The increased fluoroscopy time in the ECA group may be due to the search for the appropriate route for drug infusion and the shifted dominance between ICA and ECA caused by unstable hemodynamics (24). We observed that eyes in the ECA group have a higher proportion of cataracts, which may be related to a higher level of radiation exposure of ECA catheterization. Obesso et al. pointed out that high-dose radiation is a high-risk factor for cataracts in superselective IAC, and over 8 cycles may cause radiation exposure to exceed the onset threshold (26). However, due to the limited cases of cataract, the complex pathogenesis of cataract, as well as the proof, given by the previous study (24), that the absorption in both lens after 6 IAC cycles was still far below the onset threshold (eyes which developed cataract all received 4 IAC cycles, 3 eyes in ECA group all received only one cycle of MMA catheterization), it is hard to decide that the increased radiation in ECA group is responsible for cataract. And we will further investigate the different proportion of cataracts between the two groups in a wider population in the future.

The current research still has several limitations. First, it is a single-center retrospective study presenting the IAC treatment experience in the Han people, which may lead to existence of statistical bias. Second, ECA is an alternative to the OA route, which limits the number of such cases available for study, and too few cases may affect the statistical significance.

## Conclusion

IAC has been the main globe-preserving treatment strategy for intraocular retinoblastoma in the past decade. However, in some cases, surgeons may face technical challenges. When a satisfactory therapeutic effect cannot be achieved via OA catheterization, the alternative route of the ECA system should be considered. This alternative route is as effective as the traditional OA route.

## Data Availability Statement

The original contributions presented in the study are included in the article/Supplementary Material, further inquiries can be directed to the corresponding author/s.

## Ethics Statement

The studies involving human participants were reviewed and approved by the Ethics Committee of the Ninth People's Hospital affiliated with Shanghai Jiao Tong University School of Medicine (Number: SH9H-2020-T331-1). Written informed consent from the participants' legal guardian/next of kin was not required to participate in this study in accordance with the national legislation and the institutional requirements.

## Author Contributions

XF, RJ, and JF: conception and design. SJ, XW, and MZ: collection and assembly of data. All authors data analysis and interpretation, manuscript writing, final approval of manuscript, accountable for all aspects of the work, contributed to the article, and approved the submitted version. All authors contributed to and revised the final manuscript.

## Conflict of Interest

The authors declare that the research was conducted in the absence of any commercial or financial relationships that could be construed as a potential conflict of interest.
