# Supplementary material for: Comparison of Intra-Arterial Chemotherapy Efficacy Delivered Through the Ophthalmic Artery or External Carotid Artery in a Cohort of Retinoblastoma Patients
Source: Front Med (Lausanne). 2021 Jun 11;8:658305. doi: 10.3389/fmed.2021.658305 (PMC8225945; doi:10.3389/fmed.2021.658305)
Supplement: Supplementary file 1 [file Table_1.docx]

Supplementary Table 1. Drug regimens

| Drug | Dosage(/cycle) | Total Volume(ml) |
| --- | --- | --- |
| Melphalan* |  |  |
| 0-3 month(s) old | 0.5mg/kg | 15 |
| 4-6 month(s) old | 3.5mg | 21 |
| 6-12 month(s) old | 4.0 mg | 24 |
| 12-36 month(s) old | 5.0 mg | 30 |
| ＞36 month(s) old | 7.5 mg | 45 |
| Topotecan | 1mg | 30 |
| Carboplatin | 20mg | 30 |

* Melphalan was used in each cycle, along with topotecan in each odd-number cycle and carboplatin in each even-number cycle.
